# Deepfakes, Fake Barns, and Knowledge from Videos

**DOI:** 10.1007/s11229-022-04033-x

**Published:** 2023-01-23

**Authors:** Taylor Matthews

**Affiliations:** Department of Philosophy, University of Nottingham, Nottingham, England

**Keywords:** Deepfakes, Knowledge, Environmental luck, Epistemic risk, Cognitive ability

## Abstract

Recent develops in AI technology have led to increasingly sophisticated forms of video manipulation. One such form has been the advent of deepfakes. Deepfakes are AI-generated videos that typically depict people doing and saying things they never did. In this paper, I demonstrate that there is a close structural relationship between deepfakes and more traditional fake barn cases in epistemology. Specifically, I argue that deepfakes generate an analogous degree of epistemic risk to that which is found in traditional cases. Given that barn cases have posed a long-standing challenge for virtue-theoretic accounts of knowledge, I consider whether a similar challenge extends to deepfakes. In doing so, I consider how Duncan Pritchard’s recent anti-risk virtue epistemology meets the challenge. While Pritchard’s account avoids problems in traditional barn cases, I claim that it leads to local scepticism about knowledge from online videos in the case of deepfakes. I end by considering how two alternative virtue-theoretic approaches might vindicate our epistemic dependence on videos in an increasingly digital world.

## Introduction

A prominent view amongst many contemporary epistemologists is that knowledge is a kind of successful cognitive performance. Just as we evaluate non-epistemic performances by reference to an agent’s particular competences or abilities, this view holds that we evaluate the status of our cognitive performances (beliefs) by appeal to reliably truth-conducive cognitive dispositions – cognitive abilities or *epistemic virtues*.^1^ This forms the central plank of any virtue-theoretic account of knowledge.^2^ According to such accounts, S knows that *p* iff S’s true belief is true *because of* cognitive ability or epistemic virtue.^3^

A notorious difficulty for virtue-theoretic accounts of knowledge is cases like the following:

**FAKE BARN**: Henry and his son are driving through a part of the countryside filled with fake barn façades. As they’re driving, Henry says to his son: “there’s a barn over there”. Unbeknownst to Henry, the barn he looks at is the only real one amongst the fakes (Goldman, due to Ginet, 1976). Does Henry *know* that the barn is real?

**TWIN-EARTH**: James and Janette live on Earth and Twin-Earth. All the liquid in their global environments is water, and so they form a high number of true water-beliefs in this environment and across close possible worlds. Moreover, all the liquid in their local environments is water, so that when Janette forms the belief that she sees water her belief is true. Unknown to Janette, an indistinguishable liquid – non-water – is abundant in her regional environment but not in James’, and so she could very easily have thought she was looking at water when it was actually non-water (Kallestrup & Pritchard, 2014).^4^ Does Janette *know* she’s looking at water?

These cases are paradigm instances of *environmental epistemic luck*, and many epistemologists accept that knowledge is incompatible with this sort of luck.^5^ At their core, they embody what Duncan Pritchard (2017) has called ‘negative epistemic dependence’: that is, despite one’s belief being formed because of cognitive ability – and hence being in the market for knowledge – environmental factors beyond one’s control curtail this possibility.^6^

To alleviate this problem, Pritchard has long argued that virtue-theoretic accounts of knowledge should be supplemented with a further, sufficient *safety* condition (2012a, 2012b, 2017, 2020). For many years, he advocated for what he called *anti-luck virtue epistemology*, which supplemented cognitive ability with an ‘anti-luck’ safety condition on knowledge that sought to remedy the apparent tension between epistemic luck and cognitive ability. More recently, however, Pritchard has changed tact by recasting his preferred safety condition in terms of epistemic *risk*. On this view, a true belief is subject to knowledge-undermining epistemic risk if there is a close possible world in which that belief is formed on the same basis, but the belief would turn out *false* (2016b, 2017, 2020). For a true belief to amount to knowledge, then, not only must it be formed because of cognitive ability but it must not be subject to the kind of epistemic risk above. This is the crux of Pritchard’s *anti-risk virtue epistemology* (2017, 2020). 

My aims in this paper are twofold. The first is to demonstrate that there is a close structural relationship between the sort of cases above and what we might refer to as ‘digital fake barn’ cases. The cases I have in mind are ‘fake barn’ style cases in the sense that they exhibit the same kind of environment luck/risk as more traditional cases; however, they are ‘digital’ insofar as they draw on emerging developments in artificial intelligence, and specifically *deepfake* technology.^7^ A deepfake is an AI-generated video that depicts states of affairs that never happened, and they have grown both in popularity and sophistication over recent years.^8^ Given that barn cases have long-posed a challenge for virtue-theoretic accounts of knowledge, I contend that a similar challenge emerges in connection with digital barn cases. My second aim, then, is to consider how Pritchard’s anti-risk virtue epistemology meets this challenge. While Pritchard’s account avoids difficulties in more traditional cases, I argue that it leads us to local scepticism about knowledge from online videos when tackling digital barn cases.^9^ I end by briefly considering the prospects of two other virtue-theoretic approaches for our epistemic dependence on videos in an increasingly online world. 

The plan is as follows. In Sect. 2, I start by briefly unpacking Pritchard’s account and rehearse how it deals with the traditional cases of environmental epistemic luck above. In Sect. 3, I turn to deepfakes and construct a set of cases that I take to be analogous to the paradigm examples above. In doing so, I defend the claim that these cases are structurally analogous. With this established, I argue in Sect. 4 that the epistemic risk present in the deepfake cases is sufficient to trigger Pritchard’s anti-risk condition, not only causing his account to withhold knowledge as predicted but having the further implication of curtailing our broader claims to knowledge from online videos more generally. Finally, in Sect. 5, I consider two possible solutions to dealing with digital barn cases and knowledge from videos going forward. 

## Anti-risk virtue epistemology

According to Duncan Pritchard (2012a, 2012b, 2017, 2020), an important reason why many virtue-theoretic accounts of knowledge struggle to respond to the sort of cases above is because they rely solely on what he calls the ‘ability condition’. This condition emerges from a prior intuition about abilities more generally. Take somebody who is situated in reasonably good football-playing conditions. If they reliably score penalties whilst in these conditions, we typically laud their footballing abilities. Were they asked to score penalties in the midst of a monsoon, though, we would not likely attribute any failure to replicate their prior reliability to a lack of footballing ability; rather, we would attribute it to the abnormal conditions in which they were asked to play.^10^

A similar thought underpins the ability condition. When an agent’s perceptual faculties reliably afford them true beliefs, there is a sense in which this faculty is operating as a kind of cognitive ability the result of which is a successful cognitive performance – knowledge.^11^ The problem with relying solely on the ability condition, as Pritchard sees it, is that it gives the wrong verdict in the cases above. Specifically, it grants knowledge to the agents because they seemingly exercise cognitive ability in the formation of their true beliefs. Yet, it seems clear that neither agents really do know the respective propositions in question. To avoid this, Pritchard has long argued that the ability condition needs supplementing with a sufficient *modal* condition on knowledge. The modal condition of interest to him is a form of basis relative safety, roughly according to which a belief cannot too easily have *failed to obtain* in close possible worlds, so long as the belief was formed on the same basis as in the actual world.^12^ This formed the crux of this earlier *anti-luck virtue epistemology* (2012a, 2012b), according to which knowledge involves *safe* cognitive success, where the safety of one’s cognitive success is at least significantly (but need not be primarily) attributable to cognitive ability. 

In recent years, Pritchard has changed tact slightly, moving away from his anti-luck condition and largely replacing it with an *anti-risk* condition. Part of the motivation for this theoretical shift is his observation that our interests in excluding veritic epistemic luck from our evaluations often stem from our interests in excluding veritic epistemic *risk*. If a bullet flies past my head, for example, then I am lucky that it did not hit me. Equally important, though, is the fact that I was at risk of being hit in the first place. As Pritchard notes, a key difference between luck and risk is that the former typically focuses on the general *non-obtaining* of an event – that the bullet didn’t hit me – whereas the latter focuses on a specific risk event that we want to avoid – that I was almost shot dead.

Transferred to the epistemic domain, a belief is subject to veritic epistemic risk if there are close possible worlds in which the same basis for one’s true belief leads one to arrive at a false belief (2016b). As Pritchard admits, this construal of epistemic risk means that his anti-risk condition is not a standard safety condition, at least on his rendering. Whereas his anti-luck condition flagged close possible worlds in which an agent *failed to form a true belief* on the same basis as in the actual world, his anti-risk condition generates a refined sense of safety that explicitly flags close modal possibilities in which agents form *false beliefs.*

To see this in a better light, consider Pritchard’s (2020: 210) example of a student who has a sound memorial basis for believing that the Battle of Hastings was in 1066. Imagine, though, that while the student is confident in their belief in the actual world, there are close possible worlds in which they are disposed to doubt themselves. For example, it is just a fact about their psychology that the student’s confidence levels are quite variable, despite having no actual epistemic basis for less confidence in their belief. As Pritchard warns, there are now close possible worlds in which the student retains the same basis for the belief (remembering that the Battle of Hastings was in 1066) but now fails to *form* that belief because of their lack of confidence. Given that the student fails to form the same belief on the same basis across close possible worlds, Pritchard’s anti-luck condition withholds knowledge from the student, which he sees as incorrect. By moving to an anti-risk condition that explicitly focuses on the modal possibility of forming false beliefs, Pritchard argues that his revised safety condition better responds to the sort of case above. To recap, then, a belief is safe from epistemic risk so long as there are no close modal worlds in which the same basis for belief leads one to form *false beliefs* (2016b: 564, 2020: 210).

When fused with the ability condition, Pritchard’s anti-risk condition gives rise to what he calls *anti-risk virtue epistemology*. On this view, knowledge involves true belief that is significantly attributable to cognitive ability *and* safe from veritic epistemic risk. I will have more to say about Pritchard’s anti-risk virtue epistemology in Sect. 4, but this much should suffice to demonstrate how it deals with cases like FAKE BARN and TWIN EARTH. In both cases, we see that Henry and Jannette form their true beliefs because of cognitive ability but there are close modal worlds in which the same basis for their beliefs would lead them to form false beliefs. So, while Pritchard thinks it’s correct that both agents are cognitively successful because of ability, he maintains that their true beliefs are nevertheless unsafe because they are too epistemically risky. As a result, Henry and Janette’s true beliefs trigger Pritchard’s anti-risk condition and therefore both lack knowledge. So far, so good for anti-risk virtue epistemology.

## Deepfakes and digital fake barns

In the previous section, I introduced Pritchard’s anti-risk virtue epistemology and illustrated how it deals with cases like FAKE BARN and TWIN EARTH. I now want to introduce a second pair of cases that I referred to above as ‘digital fake barns’. My immediate aim is to show that these kinds of cases generate an analogous level of epistemic risk to more traditional fake barn cases. In order to set this up, then, it will help to first say more about the specific kind of videos that underpin these cases: *deepfakes*.

Deepfakes are ultra-realistic videos capable of depicting people doing and saying things they never did. Recent examples include deepfakes of Queen Elizabeth II’s annual Christmas Address (Channel 4, 2020), Tom Cruise, Morgan Freeman, and Barack Obama.^13^ On this rough characterisation, of course, deepfakes seem to be on par with more traditional forms of image manipulation such as CGI or Photoshop. If deepfakes and CGI footage alike can lead us to form false beliefs from a video, then do we have any reason to think that the former is in any way more problematic than the latter, at least from an epistemic perspective? Perhaps as Keith Raymond Harris (2021) puts it, the epistemic concerns with deepfakes are ‘overblown? In some respects, Harris is correct; currently, the time taken to create a sophisticated deepfake is usually longer than CGI footage or a Photoshopped picture, and because they are a relatively new technology the quality of deepfakes can vary significantly. 

An initial problem with Harris’ concern, though, is that it relies on deepfake production remaining at its current pace and quality. This, however, is far from certain. Indeed, the very name *deep*fake alludes to this prospect. Deepfake is a portmanteau of *deep* learning and *fake*: the deep learning corresponds to the deep AI ‘neural networks’ that generate the videos, and the fake element speaks for itself. Most deepfakes are created using a so-called Generative Adversarial Network (GANs), which consist of two algorithms called the ‘generator’ and the ‘discriminator’ (Mirsky and Lee, 2020: 7:7). In short, the generator creates a video based on an initial input of images and videos that try to trick the discriminator, while the discriminator works to determine whether the sample is real. Eventually, the GAN manufactures a highly authentic video, capable of mimicking the voice, mannerisms, facial expressions, and speech inflections of one person before superimposing them onto another (Mirsky and Lee, 2020). It is this aspect of deepfakes that arguably sets them apart from more traditional means of image manipulation. Unlike CGI or Photoshop, a central feature of deepfakes is that they actively self-monitor, improving the perceptual quality of the targets they depict in a more and more sophisticated manner.

Not only can the perceptual quality of a deepfake be ‘trained’, so to say, but so too can their audio quality. In recent years, researchers at Princeton University have collaborated with Adobe to create ‘VoCo’ technology, which allows video-creators to alter the content of an audio recording by simply typing words into a transcript (see, Jin et al., 2017; Rini, 2020: 6).^14^ After analysing voice samples of a target speaker, VoCo algorithms synthesise what the speaker’s voice would sound like were they to say the things written into the transcript. Accordingly, purveyors no longer need to rely on compelling voice acting to create convincing fakes. Unless there are defeating reasons to suspend judgement, our initial response will be to take the testimony we hear from a deepfake at face value as we do with regular testimony, particularly if the deepfake generates the exact voice of the target speaker.^15^

I should note that since deepfakes have mostly appeared on the internet via recorded videos, these are the kind of videos that have been subject to most manipulation. That said, deepfakes are not exclusive to the internet nor to recorded videos; an increasing number of deepfake Apps and programmes work in real-time to depict their targets doing and saying things. A sample of these include Zao, Reface, Wombo, DeepFace Lab, FaceApp, Deepfakes Web and My Heritage. As the technology improves and their remit widens, then, it is plausible that virtually any format of video could be subject to deepfake manipulation. If this is anything to go by, it looks like we could be edging closer to digital fake barn cases becoming somewhat of a reality. That is, we may well find ourselves struggling to distinguish between authentic videos and deepfakes, just as Henry and Janette struggle in FAKE BARN and TWIN-EARTH. In fact, scenarios like this are not as far-fetched as it might initially seem. Consider the following cases:

**SCAN**: Derek is a radiologist tasked with identifying a batch of scans for lung cancer tumours. He meticulously looks over one of his patient’s scans on a computer screen in his well-lit office. Unknown to Derek, a computer hacker has intercepted the other scans in the batch and added deepfake tumours to them. As it turns out, Derek looks at one of the genuine videos and correctly concludes it is tumour-free.^16^

**ALGORITHM**: Casey and Adam are twins who enjoy watching YouTube videos. All the videos they watch in their local (actual) environments are genuine, so both form a high number of true beliefs. All the videos in their global environments, moreover, are genuine so again they would form true beliefs were they to watch them. In Adam’s regional environment, all the videos he *could* causally interact with are genuine and so he would form true beliefs were he to watch them. Unfortunately for Casey, the videos that she would causally interact with in her regional environment are indistinguishable deepfakes – the YouTube algorithm suggests these to her, and so she watches them thinking they are genuine. Unlike Adam, then, Casey could very easily form false beliefs from the videos she watches.

In SCAN, it is not the case that deepfakes lead Derek to form a false belief; on the contrary, it is clear that he exercises his perceptual faculties and forms a true belief about the video scan *in virtue of* his reliable perception. Accordingly, we can establish that Derek’s true belief is correctly attributable to cognitive ability despite the prevalence of the deepfake scans. But just like in FAKE BARN, it seems mistaken to conclude that Derek *knows* that this particular video scan is tumour-free. Despite Derek and Henry being well-situated and in the correct shape to exercise their perceptual faculties, it is equally true that both could easily have formed false beliefs – by picking another patient’s scan record and looking at another barn façade, respectively. It follows, therefore, that SCAN generates an analogous level of environment epistemic risk to FAKE BARN.

What’s more, ALGORITHM resembles TWIN-EARTH insofar as it holds the initial basis-relative conditions fixed across Casey and Adam’s actual environments, such that they form beliefs in the same way across the board. Since they are twins, we can work on the assumption that they share similar physiological profiles like James and Janette. In line with TWIN EARTH, though, ALGORITHM changes the close modal environments in a way that would lead Casey but not Adam to form false beliefs upon exercising her cognitive abilities. For Casey, the YouTube algorithm ensures that her regional modal environment contains indistinguishable deepfakes, and thus there exists a close possible world in which she exercises her cognitive abilities but could very easily have formed a false belief on the same basis.

Part of what makes SCAN and ALGORITHM interesting is that they are more feasible than fictional examples like FAKE BARN and TWIN-EARTH. Due to their fictional nature, a number of studies have found that some people are inclined to grant knowledge in cases like FAKE BARN (Bergenholtz et al., 2021, Colaço et al., 2014, Sosa, 2007). As Sosa (2009: 107) notes, when it comes to reading fictional cases, we often ‘import a great deal that is not explicit in the text’. So, the less fictional or artificial a case is, the less details there are to import or ‘fill in’. Given that SCAN and ALGORITHM draw on a real, emerging technology, our intuitions about knowledge-ascriptions in these cases will hopefully be subject to less interpretation and hence be more convincing than their fictional counterparts. As such, there is less reason to think that people will grant knowledge in these digital fake barn cases. 

Perhaps more importantly, though, deepfakes are a *relevant alternative* to videos in the sense that they are becoming increasingly prominent features of our digital environments (Dretske, 1970; Nozick, 1981). Compare this with the environments of FAKE BARN and TWIN-EARTH. In both cases, the agents struggle to discriminate between the genuine and fake perceptual objects because they are in abnormal environments, and hence the perceptual objects are *irrelevant* alternatives. What makes this the case is that they are both probabilistically unlikely and/or would fail to obtain in close modal worlds. However, when it comes to our cases above, this sort of response cannot so easily be invoked due to the increasingly digitalised world in which we live. Regardless of how one frames relevant alternatives (in probabilistic or modal terms), it is not unreasonable to suggest that SCAN and ALGORITHM are more relevant in the sense required for perceptual knowledge. If this is the case, then deepfakes could quite easily subject our perceptual beliefs to an unwelcome level of epistemic risk.

## Deepfakes and anti-risk virtue epistemology

Now that I have introduced a set of ‘digital fake barn’ cases, I will argue that a variation of them raises a challenge for Pritchard’s anti-risk virtue epistemology. Let’s start by briefly reminding ourselves of the core tenet of his account:

*S* knows that p if S’s true belief is significantly attributable to cognitive ability, and there are no close modal worlds in which S’s true belief could be false whilst formed on the same basis (2017, 2020).

If we work on the assumption that SCAN and ALGORITHM generate similar levels of environmental risk to FAKE BARN and TWIN-EARTH, then we can predict how Pritchard’s anti-risk virtue epistemology will treat Derek and Casey. Given that the pair could very easily have formed false beliefs on the same basis in close possible worlds, the true beliefs they do form are unsafe. As a result, both trigger Pritchard’s anti-risk condition and they thereby lack knowledge. This seems correct.

Things get more interesting, however, if we compare two further cases: SCAN* and ALGORITHM*. In these modified cases, suppose there is now only one deepfake in Derek’s batch of scans and that the YouTube algorithm only plants one deepfake into Casey’s regional modal environment. Again, suppose that both are unaware of the deepfake and it is of the same quality. Does removing the majority of the deepfakes make a difference to how Pritchard’s account might handle the cases? To begin answering this, recall that the anti-risk condition at the heart of his account is not modelled on probability but on *modality*. The reason for this is that it allows his account to nicely handle lottery cases. As Pritchard puts it, winning the lottery ‘while probabilistically farfetched is in fact modally close’ (2016b: 553). More generally, he observes that an event can be ‘modally close even when probabilistically unlikely’ and that ‘one cannot infer from the fact that an event is probabilistically unlikely…that it is therefore also modally far-off’ (2016b: 553).^17^ The point at stake, then, is that even if we think that SCAN* and ALGORITHM* are probabilistically unlikely, this has little bearing on their modal distance. 

What ultimately determines whether a risk event is modally close is how *similar* the world is to the one in which the event takes place (Pritchard, 2016b: 553, 2017: 2882, 2020: 209). To make this point more salient, compare the tweaked cases above with FAKE BARN*. In this modified barn case, suppose there is just one barn façade in Henry’s immediate environment, but he still looks at a genuine barn. Given that we have reduced the number of barn façades in this new modal environment, Pritchard’s anti-risk condition would track this shift because of the changes we have to make to Henry’s *actual* world. Since we have to remove almost all the fake barns in order for Henry’s true belief to be safe from epistemic risk, we are eroding the similarity of his new modal environment to the actual world he was in beforehand. In turn, FAKE BARN* becomes a modally distant world and the corresponding degree of epistemic risk bearing on Henry’s true beliefs would be almost irrelevant. Accordingly, Pritchard’s account would likely ascribe knowledge to Henry now.

Notice, though, that in SCAN* and ALGORITHM* the reverse seems to happen. By removing the number of deepfakes from Derek and Casey’s original modal environments, we arguably bring their modal worlds closer in line to our actual worlds. This is precisely because our current digital environments are not flooded with deepfakes in a way that characterises my original cases; rather, the kind of scenarios captured by the tweaked cases better reflect the nature of our actual digital environments. Put differently, the modal possibility of watching just a single sophisticated deepfake is closer to our actual world. On this basis, we can establish that the risk events of Derek and Casey watching the single deepfake are relevantly close. The upshot now, however, is that their true beliefs in SCAN* and ALGORITHM* are subject to a sufficient degree of epistemic risk. As a result, their perceptual beliefs remain unsafe and thus trigger Pritchard’s anti-risk condition. In line with his diagnosis of the original cases, then, Pritchard’s view continues to withhold knowledge from Derek and Casey.

If our intuitions about knowledge in my tweaked cases are the same as the original cases, then it would appear as though Pritchard’s account offers the correct verdict and his anti-risk virtue epistemology is in the clear. But if this is indeed how Pritchard’s view would diagnose Derek and Casey in SCAN* and ALGORITHM*, then I want to flag an implication this conclusion has for our broader claims to certain knowledge from videos.^18^Recall that the reason why Derek and Casey’s true beliefs remain subject to knowledge-undermining epistemic risk is because their modal environments in the tweaked cases are sufficiently similar to our actual digital environments. If this were not the case, after all, we could most likely disregard the epistemic risk at play because the possibility for error would be further away. But since the margin for error is a close possibility, this means that the presence of the single deepfake renders their other true beliefs unsafe. 

The problem is that this conclusion appears to generalise beyond SCAN* and ALGORITHM* to an important domain of videos that we increasingly rely on. I noted above that the most sophisticated deepfakes are currently found online or in recorded videos. At present, the probability of us watching such a sophisticated deepfake remains considerably low. Nevertheless, it is not too difficult to see how the kind of world that characterises the tweaked cases above is modally close to our actual world, especially given the similarities between the number of deepfakes in our current digital environments and that of SCAN* and ALGORITHM*. Indeed, just as deepfakes continue to develop, so too will the similarity between these two cases and our actual world. The upshot is a scenario in which the vast majority of our perceptual beliefs from online and recorded videos remain true but there exists a close possible world in which we watch a single sophisticated deepfake. Much like the tweaked cases, then, the presence of the sophisticated deepfake would render our perceptual beliefs unsafe. 

In fact, this not only applies to our perceptual beliefs but to our testimonial beliefs. As I highlighted above, deepfake technology can be used to tamper with audio recordings, making a depicted speaker say things that are typed into a transcript in their own voice. In recent years, this method of deepfake has become popular amongst fraudsters. For example, in 2020 a bank manager in Hong Kong reportedly transferred $35 million to a company director with whom he had previously spoken. Convinced by the director’s voice and legitimacy, the transfer was made. On this occasion, though, it was not actually the director speaking but fraudsters employing ‘deep-voice’ deepfakes to impersonate him (Brewster, 2021).^19^ While these kinds of deepfakes are less common than visual deepfakes, they have the propensity to generate a similar challenge: we can find ourselves listening to a genuine piece of audio and form true testimonial beliefs, yet the presence of a single sophisticated deepfake would become a gradually closer modal possibility as the technology develops and spreads. Accordingly, our testimonial beliefs from videos would be rendered unsafe by the deepfake. If one accepts the ‘no-knowledge’ verdict in SCAN* and ALGORITHM, as Pritchard’s view prescribes, then it looks as though one must also endorse local scepticism about perceptual (and possibly testimonial) knowledge from online videos, at the least.

Of course, as Pritchard (2004: 330) himself has pointed out, just because an epistemological view leads to scepticism, this is hardly an objection in and of itself since the scepticism could be warranted. While this is true, the problem with this sort of response is that it leaves us without an explanation of our epistemic dependence on online and recorded videos if they can no longer serve as a means of acquiring knowledge. In a world in which we depend ever more on such videos for information, especially after the Covid-19 pandemic, it seems uncontroversial to say that we want an epistemology that tells us how we go about gaining knowledge from a source as ubiquitous as videos, in much the same way we do for testimony. Thus, the burden of proof is on Pritchard to tell us a story of how we can navigate our epistemic lives in an increasingly digital world. 

One possibility would be to claim that my cases do not generalise to online videos because we are usually sensitive to the *sources* of video and testimony we watch and listen to. Accordingly, if a video looks dubious or a speaker vague or elusive, then we are far less inclined to trust their reliability (Pritchard, 2012a). Given that much of our perceptual and testimonial knowledge comes from established and trustworthy sites, it seems difficult to imagine how digital fake barns might be problematic.

For this objection to be viable, though, it must be the case that we *remain* in a position to trust online videos as a credible epistemic source. The first thing we can say here is that the increasingly sophisticated nature of deepfakes means that it is becoming much harder to discriminate between genuine videos and deepfakes. This goes for both our visual and auditory capacities. In the case on which SCAN is based, for instance, the radiographers were tasked with identifying the deepfake tumours amongst genuine tumours and they failed ninety percent of the time to do this. Even after being informed about the deepfakes, the radiologists still misdiagnosed the scans with deepfake tumours sixty percent of the time (Mirsky et. al., 2019). Despite being trained professionals, the participants were not able to reliably discriminate between genuine and fake video scans. 

I submit that this does not relate to online videos specifically but the task of creating sophisticated deepfakes is arguably much easier in this domain. This is especially the case once we factor in VoCo algorithms and other ‘deep-voice’ technology mentioned above. As the lead author of the research into these algorithms reported (Jin et. al., 2017: 96:10), when the synthesised words were inserted in the context of a spoken sentence, the modified sentence was ‘often perceived as indistinguishable from other sentences spoken in the same voice’. If this technology becomes widespread enough, then our sensitivity to discernible features of online media and videos seems vulnerable to deepfakes.

This last point speaks to a more general worry about the epistemic credentials of videos. It is widely held that videos and photographs provide us with *perceptual* evidence of states of affairs, and that this kind of evidence is more authoritative than, say, testimonial evidence (Cavedon-Taylor, 2013; Hopkins, 2012). The worry is that if deepfakes become increasingly sophisticated, they will lead to a sense of ‘displaced epistemic reality’, where the very possibility of an authentic video being a deepfake lingers in our minds (Rini, 2020: 8).^20^ This would not only risk jeopardising the trust we place in videos, particularly if it becomes increasingly difficult to establish the veracity of a video, but any loss of trust will in turn weaken the justificatory status of videos as a whole. As such, Pritchard cannot rely on us being continually sensitive to the kind of videos we watch.

Still, Pritchard could try to get around this conclusion by recasting his anti-risk condition along probabilistic lines. By doing so, his account could possibly evade scepticism about videos because of the low-risk probability of watching a sophisticated deepfake. Unfortunately, such a move would undermine his broader project of developing a satisfactory anti-risk epistemology. As I emphasised above, Pritchard formulates his anti-risk condition in modal terms precisely because it allows us to offer a plausible verdict in lottery cases. The problem is that if he were to recast this condition in probabilistic terms, the low probability of winning the lottery would commit his anti-risk epistemology to ascribing knowledge in these cases since the risk of one’s belief about winning turning out false would be very low. What’s more, if Pritchard were to make this move, he would have to abandon his preferred rendering of safety that allows his anti-risk virtue epistemology to circumvent the problems raised by FAKE BARN and TWIN-EARTH. In light of this, Pritchard is unlikely to pursue this avenue.^21^

Finally, Pritchard could reject the premise that sophisticated deepfakes will cause us to form false beliefs. Instead, he could claim that they will likely cause us to suspend judgement and so lead us to form no beliefs about what we watch. Recall that his anti-risk condition, unlike his earlier anti-luck condition, is designed to explicitly flag true beliefs that could turn out false in close modal worlds as opposed to worlds in which agents fail to form beliefs (2016b: 564, 2020: 210). But if deepfakes just cause us to suspend judgement and hence form no beliefs, then they would not trigger his anti-risk condition and his virtue epistemology would face no difficulties. 

For the sake of argument, let’s suppose that people will not form false beliefs from deepfakes. The problem, again, is that in an increasingly digital world, it is not a viable option to suspend judgement about videos. Given how much information videos generate and transmit, suspending judgement would not only undermine the role videos play in gaining historical knowledge (such as which figures were present at which events), but our everyday affairs, such as watching videos of holiday destinations, wedding venues, or real estate.^22^ More worryingly, if agents suspend judgment about videos and so form no beliefs, then Pritchard’s anti-risk virtue epistemology becomes redundant. It is true that agents would no longer trigger his anti-risk condition, but if they do not form perceptual beliefs from online videos to begin with, then his account cannot attribute knowledge to those agents either. While Pritchard’s anti-risk virtue epistemology might handle traditional barn cases well, then, the same cannot so easily be said for how it treats digital barn cases that are the result of deepfakes. If we want an epistemology that can account for our dependence on online videos, perhaps we are better served looking elsewhere.

## Robust and responsibilist alternatives

In this paper, I have claimed that developments in deepfake technology have the propensity to create what I refer to as *digital fake barns*, which embody the same degree of epistemic risk as traditional barn cases. I then examined whether Pritchard’s anti-risk virtue epistemology can alleviate the problems these cases might raise. The result was that his account leads us towards local scepticism about an important and increasingly valued kind of knowledge, namely knowledge from online videos. A remaining question that needs addressing is how we might go about handling the challenges presented by deepfakes and digital barn cases more generally. In what follows, I draw on two alternative virtue-theoretic approaches that could offer insights here. The first is John Greco’s (2010, 2020) robust virtue epistemology.

In his most recent work on ‘knowledge-producing abilities’, Greco (2020) explicitly opts to relativise cognitive abilities to specific modal environments, claiming that S has a cognitive ability A ‘relative to a modal environment’, just where S is ‘reliably successful, when in appropriate conditions – correct shape (Sh) and situation (Si) – within that modal environment’ (2020: 130).^23^ By *reliably successful* Greco means that an agent not only has a ‘disposition seated in inner seat (Se) to believe truths in an appropriate range of propositions R', but that they also achieve this ‘throughout that modal space’ – i.e., they are reliably disposed to cognitively succeed in the close possible worlds *relative* to the range of propositions R in question (2020: 131, 133). Second, by relativising cognitive abilities to modal environments, Greco claims that his view of cognitive ability entails a *weak* safety condition.^24^ Specifically, he claims that throughout the space of worlds defined by one’s modal environment, ‘*almost always* when S believes that p (while retaining Se, and in Sh and Si), p is true’ (2020: 134, my italics). As this suggests, Greco importantly allows cognitive abilities to be compatible with a very small range of error across the relevant modal environment.

Considering this, Greco’s robust virtue epistemology offers a different diagnosis of the digital fake barn cases considered above. In keeping with his earlier diagnoses of cases like FAKE BARN and TWIN EARTH, Greco would rule out knowledge in SCAN and ALGORITHM much like Pritchard. However, Greco’s reason for ruling out knowledge in these cases turns on a premise that Pritchard accepts, and he does not: that Derek and Casey form true beliefs on account of their cognitive abilities. On Greco’s view, the fact that neither can reliably discriminate between the genuine and the fake videos reveals that their perceptual faculties are not operating in the sort of modal environments conducive to cognitive ability. Again, this is because they cannot reliably succeed at forming true beliefs if they tried.

Unlike Pritchard’s account, however, it seems that Greco’s view would attribute knowledge to Derek and Casey in our revised cases of SCAN* and ALGORITHM*. This is due in large part to his decision to relativise cognitive abilities to modal environments, which in turn permits some false beliefs across the relevant modal space. So, while the pair lack ability in the original cases, removing the vast majority of deepfakes in the tweaked cases now means that they are far more likely to succeed at forming true beliefs in their revised modal environments. Although the presence of the single deepfake may cause them to form a false belief in their respective modal environments, it does not take away from the pair ‘almost always’ truly believing that *p*. As such, their perceptual faculties operate in a modal environment that is far more congenial to cognitive ability. This fact about them reveals that their perceptual beliefs are formed *because of* ability, and therefore that they are in the market for knowledge.

In offering this diagnosis, Greco’s view has important implications for our more general claims to knowledge from online videos. Given that his knowledge-producing abilities do not require perfect reliability across the relevant modal space, the presence of the single sophisticated deepfake does not deprive our perceptual vision the status of cognitive ability in the actual world. Therefore, when we watch videos in this modal environment, we would form the true beliefs we do *because of* our perception operating as a cognitive ability. As a result, it would seem that Greco’s robust virtue epistemology avoids the local scepticism about online videos that Pritchard’s account leads to. It should be said, of course, that if one finds this verdict counter-intuitive, then this only shows that deepfakes and digital barn cases provide accounts like Greco’s (and perhaps others) with food for thought about how we might wish to go about acquiring knowledge in an increasingly digital age. The second alternative I want to briefly consider originates in a different kind of virtue epistemology altogether. My focus in this paper has been on reliabilist virtue epistemology, but as is widely known there is also a brand of virtue epistemology that focuses on the *intellectual character* of agents. This character-based or *responsibilist* virtue epistemology is primarily concerned with the character traits and attitudes that make us good inquirers such as open-mindedness, intellectual perseverance, and intellectual humility (Baehr, 2011; Battaly, 2008; Zagzebski, 1996).^25^

In recent work, I have drawn on responsibilist virtue epistemology to develop what I call a well-trained *digital sensibility* (Matthews, 2022). This concept is an extension of Miranda Fricker’s (2007) earlier notion of a well-trained testimonial sensibility, which is roughly a perceptual and affective sensitivity to the epistemically salient features of a speaker’s situation (their trustworthiness and the sincerity and competence of their testimony).^26^ Accordingly, the notion of a well-trained digital sensibility appeals to similar considerations about the trustworthiness, sincerity, and competence of online content. The idea is that when we are confronted with certain videos, images or websites, a well-trained digital sensibility enables agents to ‘just see’ the content as trustworthy. Much like its testimonial counterpart, this will turn on features relating to the competence and sincerity of the online content itself (2022: 79–81). 

Although I cannot fully reconstruct the notion of a well-trained digital sensibility here, I shall focus on one aspect that might aid us in detecting deepfakes. Unlike Fricker’s testimonial counterpart, digital sensibility does not establish the sincerity and competence of online content through empathy. Instead, it depends largely on fine-tuning a sort of healthy scepticism towards such content, which we can think of as an evidentially sensitive stance between gullibility and close-mindedness (Le Morvan, 2011: 98, 2019). The success of this fine-tuning, I contend, centrally involves cultivating various intellectual character virtues, ranging from intellectual perseverance in fact-checking online content to intellectual humility in recognising that our credibility assessments of online videos might not always be watertight. As we interact more and more with different sources of online content, evaluating the competence in their presentation or the sincerity of the claims they make, the hope is to gradually build up a catalogue of past experiences that can inform one’s approach to an array of digital media. The result is that those with a well-trained digital sensibility will be better placed at discerning trustworthy from untrustworthy online content, including deepfakes. Here, I think the responsibilist character virtues will have an indispensable role to play going forward.

## Conclusion

I want to end by drawing three broad conclusions from the paper. The first is that developments in deepfake technology risk creating a number of what I have called ‘digital fake barn’ cases, and I set out several here. In light of this, I suggest that epistemologists now pay greater attention to the application of deepfakes and their potential epistemic costs. The second is that deepfakes and digital barn cases raise important challenges for our claims to knowledge from online videos. I articulated this challenge by appeal to Duncan Pritchard’s recent anti-risk virtue epistemology. The third is that these challenges generate interesting questions about how we can go about dealing with deepfakes and digital barn cases more broadly. While I could only offer two brief possibilities here, the hope is that epistemologists now accept the call.^27^

## Footnotes

1 As this suggest, my focus here is on ‘reliabilist’ virtue epistemology as opposed to ‘responsibilist’ or character-based virtue epistemology (Baehr, 2011; Zagzebski, 1996). However, I briefly touch on virtue responsibilism in Sect. 5 and so make the relevant distinction apparent.

2 This forms the central plank of both ‘robust’ and ‘modest’ virtue epistemologies. As I explain below, the major difference is that the latter camp think cognitive ability alone is insufficient for knowledge. For important contributions to ‘robust’ virtue epistemology see, e.g. Broncano-Berrocal, (2017b, 2018), Carter (2016), Greco (2010, 2020), Nevarro (2015), Sosa (2007, 2011, 2015), and Turri (2011, 2016). See Kelp (2013), Kallestrup and Pritchard (2013, 2014), and Pritchard (2012b, 2017, 2020) for contributions to ‘modest’ virtue epistemology.

3 The attribution relation of ‘*because of*’ differs between those who think of ability as *causally explaining* knowledge (Greco, 2012, 2020; Pritchard 2012b, 2020) and those who think of ability as *manifesting* knowledge (Kelp, 2013; Sosa, 2007, 2015; Turri, 2011). We need not adjudicate this difference here.

4 Following Kallestrup and Pritchard (2014: 339–340) we can understand ‘**local environment**’ to be where Janette and James are currently located, containing objects and properties that are the proximate cause of their current perceptual experiences. Local features relating to their perception could include, for example, distorting noise, brightness etc. We can understand ‘**regional environment**’ as neither where Janette nor James is currently located, nor where they typically form any beliefs. But it does contain properties and objects with which they could easily have causally interacted. Regional facts are ‘nearby possibilities’ but they play no causal role in their perceptual experiences on which they base their beliefs. Finally, we can understand ‘**global environment**’ to be where James and Janette are ‘normally located’, though not at present. It contains objects and properties with which they ordinarily interact. ‘Global facts’ here are not only distant perceptual possibilities, but also causally inefficacious in producing Janette’s and James’ current perceptual experiences.

5 That said, studies have suggested that non-philosophers are willing to grant agents knowledge in cases like FAKE BARN. See, e.g. Bergenholtz et al. (2021), Colaço et al. (2014), Sosa (2007), and Turri (2016, 2019).

6 Epistemic dependence can also be *positive*, where one’s cognitive agency would not normally suffice for knowledge but factors beyond one’s agency put one in a position to know. My focus here is exclusively with its negative manifestation. See Kallestrup and Pritchard (2013, 2014) and Pritchard (2016a) for relevant discussions.

7 ‘Digital fake barns’ need not be restricted solely to videos as is the case here. They might reasonably emerge in connection with technologies that alter the audio of speakers, and so reflect cases of a reliable testifier amongst unreliable sources of testimony.

8 There is relatively little work on the epistemology of deepfakes. Notable exceptions include Fallis (2020), Rini (2020), Harris (2021) and Matthews (2022).

9 Since deepfakes feature most prominently in online spaces and via recorded videos, it is these formats of video with which I am largely concerned here. As I note below, though, increasingly sophisticated deepfakes have appeared in real-time video recordings and so their implications may eventually have a greater remit than argued for here.

10 See Greco (2008, 2020) and Sosa (2015) for discussions on the nature of epistemic ability/competence and abnormal conditions.

11 Though, see Horst (2021) for an argument against conceiving of cognitive abilities on the model of skill or competence.

12 For variations and discussion of safety in epistemology, see Luper (1984), Sainsbury (1997) Sosa (1999a), Williamson (2000), and Pritchard (2005).

13 To watch these highly sophisticated deepfakes, see, for example, https://www.youtube.com/watch?v=oxXpB9pSETo&ab_channel=DiepNep,https://www.youtube.com/wa tch?v = AmUC4m6w1wo&ab_channel = BBCNews, https://www.youtube.com/watch?v=i OIoU9U9gZg &t=2s&ab_channel=TheTelegraph, https://www.youtube.com/watch?v=CDMVaQOvtxU&ab_channel=TODAY.

14 A demonstration of this technique is available at < https://www.youtube.com/watch?v=RB7upq8nzIU > 

15 The allows us to see how ‘digital fake barns’ can function as cases of ‘bad testimony’, where an agent forms a true belief on the basis of an informant’s testimony, but unknown to them the informant is the only reliable one in a community of unreliable informants. In short, they are cases where the agent’s true testimonial belief could easily turn out false across close possible worlds. I return to this point below.

16 This example is inspired by real events conducted at a hospital in Israel. See Mirsky et.al (2019). While the scans in their study were primarily image-based, the injected deepfakes were 3D videos.

17 As Pritchard notes, the orthodox accounts of risk are modelled on probability as opposed to modality, but he believes that modal risk better captures probabilistic risk. For more on this, see Pritchard (2015b). For probabilistic accounts of risk, see Hansson (2014, 2004).

18 I thank an anonymous reviewer for urging me to explicitly clarify the implications that Pritchard’s account leads to if it does withhold knowledge in these two tweaked cases.

19 For similar cases, see Damiani (2019) and Union Bank (2022).

20 Of course, this is only the case *insofar* as first-hand, direct testimony is unavailable. However, since videos increasingly provide ‘second-hand’, indirect testimony, this is where Rini’s worries would seem applicable.

21 A further consideration that Pritchard offers in favour of his modal anti-risk condition is that it allows us to make clear discriminations between risk events that have *prima facie* similar probabilities, but where one event is seemingly closer away in terms of possible worlds (2015: 442).

22 I thank Dan Cavedon-Taylor for raising this point.

23 In earlier work, Greco (2001: 60, 66, 67) claims that we ought to understand cognitive abilities by reference to ‘possible world semantics of modal logic’, and that cognitive ability is a ‘function of S’s rates of success *across close possible worlds*’. More recently, he suggests that ‘across the set of *relevantly close worlds*’ W, where S is in the correct conditions C within an environment E, S has a high rate of achieving cognitive success (2008: 64, my italics).

24 It is worth stressing that Greco does not think the ability condition needs supplementing with an additional safety condition. The purported ‘weak’ safety condition he speaks of is built into the ability condition itself, such that a belief formed via cognitive ability would not too easily turn out false across close the relevant modal environment if formed on the same basis (2020).

25 Zagzebski’s (1996) *Virtues of the Mind* is the most developed attempt at offering a responsibilist analysis of knowledge.

26 On Fricker’s view, this sensitivity is also grounded by (1) good judgement that is uncodifiable i.e. it will not be rule-based, (2) be intrinsically motivating, and (3) be reason-giving i.e. generate reasons to act in certain ways (2007: pp. 72–80). For ease of discussion, I only refer to its perceptual and affective dimensions here.

27 I would like to thank audiences at the University of Nottingham and Cardiff University, respectively, for valuable feedback on earlier iterations of this paper. I would also like to extend my upmost thanks to J. Adam Carter, Dan Cavedon-Taylor, Ian James Kidd, Michael Hannon, and two anonymous referees at *Synthese* for their constructive feedback that helped improve this paper. This paper was written whilst in receipt of a Midlands4Cities Doctoral Training Scholarship, for which I very grateful.
